# Oxide Derived Copper for Electrochemical Reduction of CO_2_ to C_2+_ Products

**DOI:** 10.3390/nano12081380

**Published:** 2022-04-18

**Authors:** Anum Zahid, Afzal Shah, Iltaf Shah

**Affiliations:** 1 Department of Chemistry, Quaid-i-Azam University, Islamabad 45320, Pakistan; anumzahid08@gmail.com; 2 Department of Chemistry, PMAS Arid Agriculture University, Rawalpindi 46300, Pakistan; 3 Department of Chemistry, College of Science, United Arab Emirates University, Al Ain P.O. Box 15551, United Arab Emirates

**Keywords:** carbon dioxide reduction reaction (CO_2_RR), oxide derived copper (ODCu), faradaic efficiency, C_2+_ products

## Abstract

The electrochemical reduction of carbon dioxide (CO_2_) on copper electrode derived from cupric oxide (CuO), named oxide derived copper (ODCu), was studied thoroughly in the potential range of −1.0 V to −1.5 V versus RHE. The CuO nanoparticles were prepared by the hydrothermal method. The ODCu electrode was used for carbon dioxide reduction and the results revealed that this electrode is highly selective for C_2+_ products with enhanced current density at significantly less overpotential. This catalyst shifts the selectivity towards C_2+_ products with the highest Faradaic efficiency up to 58% at −0.95 V. In addition, C_2_ product formation at the lowest onset potential of −0.1 V is achieved with the proposed catalyst. X-ray diffraction and scanning electron microscopy revealed the reduction of CuO to Cu (111) nanoparticles during the CO_2_ RR. The intrinsic property of the synthesized catalyst and its surface reduction are suggested to induce sites or edges for facilitating the dimerization and coupling of intermediates to ethanol and ethylene.

## 1. Introduction

The anthropogenic carbon emissions and rise in global temperatures has increased the demand for energy production. Renewable energy sources are continuing to progress for greater advantages, but long-term energy storage remains a challenge that must be addressed to meet the global energy demand. In this regard, CO_2_ reduction not only meets the requirement of energy storage but also helps in mitigating the anthropogenic carbon dioxide emission. Energy storage in chemical bonds has several advantages as compared to battery storage in terms of high energy density, transportability, and enhanced safety [1,2,3,4,5,6,7]. When coupled with carbon capturing technology, the carbon dioxide reduction offers a solution for balancing the natural carbon cycle. The electrochemical reduction of carbon dioxide involves multiple electron transfer steps depending upon the electrocatalysts. The energy barrier to CO_2_ reduction is lowered by an electrocatalyst by a mechanism involving stabilization of the intermediates in the multistep electrochemical reduction process. Among all the CO_2_ reduction products; ethene, ethanol and propanol have higher volumetric energy density and commercial values. Various electrodes have been used for this purpose, especially Cu, which is capable of catalyzing the electrochemical reduction of CO_2_ to highly reduced products (any product requiring the transfer of >6 electrons in an aqueous solution) at near-neutral pH in high yields [8,9,10,11,12].

2CO_2_ + 8H_2_O + 12e → C_2_H_4_ + 12OH^−^

2CO_2_ + 9H_2_O + 12e → C_2_H_5_OH + 12OH^−^

C_2_ products are commercially more important as compared to C1 products and can act as building units for the synthesis of oxygenates, polymers and long chain hydrocarbon fuels. However, until now, the reported highest Faradaic efficiency of the C_2_ products is lower as compared to C_1_ products (close to 100%) [12,13,14,15]. This may be due to the complex reaction pathways involved in C_2_ products formation as a much higher kinetic barrier of the C–C coupling step may be responsible for decreasing energy efficiency. Generally, a higher potential is required for the reduction of compounds containing C=O than those containing C―H and C―OH groups. The CO_2_RR process involves three main steps. The first step is the adsorption and bonding of CO_2_ with surface atoms of the catalyst. The second step is the activation and then the reduction of CO_2_ involving electron/proton transfer processes. The third step is the desorption of the products from the catalyst surface. Copper has a unique catalytic property of producing hydrocarbons and alcohols with promising Faradaic efficiency [15,16,17,18,19,20]. Copper is arguably the best-known electrocatalyst for electrochemical CO_2_ reduction as it is capable of electrochemically converting CO_2_ into more than 30 different products including hydrocarbons and alcohols. Cu metal increases the CO dimerization process and hence facilitates the formation of C_2_ products. Product distribution mainly depends upon the surface geometry and morphology of Cu surfaces, as well as on applied potential and electrolytes. Oxide derived copper catalysts have enhanced selectivity for C_2_ products. Their selectivity depends on the oxidation state and surface morphology of copper. It is believed that the reduction of copper oxide catalyst can induce undercoordinated sites, grain boundaries and rough surfaces as catalytically active sites. In recent years, many investigations have led to the development of many efficient metal-based electrocatalysts for CO_2_RR [20,21,22,23]. Nano catalysts such as nanoparticles, nanowires, nanotubes and nanofoams have shown improved selectivity and efficiency over bulk materials [20,21,22,23,24,25,26]. Nano catalysts derived from copper oxides have shown better selectivity towards ethylene and ethanol (C_2_ products) at lower overpotentials. Despite these remarkable results of oxide derived copper nanoparticles for CO_2_RR, the mechanism is still unclear. It is believed that grain boundaries may be the catalytic active sites induced by the oxygen derived copper species. The other reason could be the higher current densities on the defect sites that increase the local pH, which may alter the reaction pathway in favor of ethanol and ethylene. It is generally accepted that Cu oxides are completely reduced at a very negative potential (−1.0 V vs. RHE) and the reaction only occurs on metallic copper. Copper catalysts with different morphologies can be obtained from various synthetic methods such as annealing, electrodeposition, the hydrothermal method, chemical treatment, and colloidal synthesis [15,16,17,18,19,20,21,22]. The current study presents the performance of hydrothermally produced surfactant coated CuO nanoparticles towards CO_2_RR. CuO NPs in the presence of triton were synthesized and the resulting catalytic property towards CO_2_ reduction was evaluated using gas chromatography and NMR spectroscopy. The effects of electrolyte, crystal orientation and nanoparticles size were examined on the product formation of ethene, ethanol and propanol.

## 2. Materials and Methods

### 2.1. Experimental Section

#### 2.1.1. Chemicals

Copper acetyl acetone (99.9%), triton X-100 (99.0%), ammonium carbonate, deuterium oxide (99.9% purity), NaOH, and Nafion (5 wt%) were purchased from Sigma-Aldrich; sodium bicarbonate (ACS grade), potassium bicarbonate, acetone (99.9%) and dimethyl sulfoxide (99.9%) were purchased from Merck. Nitrogen (99.9%) and carbon dioxide (>99.9%) gases were purchased from Air Liquide, Melbourne, Australia. High purity water obtained from a Milli-Q water purification system was used for all the aqueous sample preparations.

#### 2.1.2. Preparation of CuO Nanoparticles

CuO nanoparticles were synthesized by the reported co-precipitation method [24]. Firstly, 0.5 M solution of copper acetate (Cu(CH_3_COO)_2_) was prepared. Then, 2 mL of the Triton X-100 was added into it drop wise followed by stirring for 40 min. Then 0.5 M solution of ammonium carbonate ((NH_4_)_2_CO_3_) was added in the solution drop wise and stirred up to 40 min at 60 °C. In order to maintain the pH of the solution between pH 10–11, 1 M solution of NaOH was added drop wise followed by stirring of the solution up to 1.5 h at 90 °C. The precipitate was then centrifuged at 13,000× *g* rpm and washed several times with deionized water and ethanol. CuO nanoparticles were then dried at 80 °C over night and followed by grinding the precipitates into fine powder.

#### 2.1.3. Preparation of Glassy Carbon Plate/Electrodes

Prior to experiments, a glassy carbon electrode was polished with alumina powder and then washed with distilled water, followed by drying under a stream of N_2_. The glassy carbon electrode with a geometric area of 0.07 cm^2^ was used for cyclic voltammetric experiments. The GCE with a geometric area of 1 cm^2^ was used for bulk electrolysis.

#### 2.1.4. Instruments for SEM and XRD

Scanning electron microscopic images were obtained using ZEISS EVO 40 (Oberkochen, Germany). For analysis of the morphology of nanoparticles, the XRD technique was employed using Bruker D8 ADVANCED power diffractometer (Bruker, Germany) with source Cu Kα radiation (λ = 0.154 nm).

#### 2.1.5. Electrochemical Instrumentation and Procedures

All electrochemical experiments were carried out at room temperature in a three-electrode system using a CHI700D electrochemical workstation (CHI Instruments, Austin, TX, USA). The CuO derived Cu coated carbon plate electrode was using as a working electrode (cathode), Pt plate as counter electrode and Ag/AgCl (3M KCl) as the reference electrode. The potential was converted to RHE scale using the formula:

E (vs. RHE) = E (vs. Ag/AgCl) + 0.190 V + 0.0586 V × pH.

Bulk electrolysis was performed in a two-compartment gas tight H-shaped cell under a CO_2_ atmosphere with the cathodic and anodic compartments separated by a porous glass frit. Prior to electrolysis, CO_2_ gas of high purity was used to saturate the solution for almost 20 min in a H type cell, which was tightly sealed with a rubber stopper.

#### 2.1.6. Analysis of the Electrolysis Products

Gaseous product was identified by Gas Chromatography (GC) in the headspace of an H-cell working electrode of a bulk electrolysis cathodic compartment. Calibration curves for C_2_H_4_ and H_2_ were built by injecting known concentrations of pure C_2_H_4_ and H_2_. The standard calibration plot was constructed by plotting the area of the peak against the amount of gases injected. Each gaseous product was identified by the retention time and quantified by the relevant calibration curve. The liquid products were characterized by 1H NMR spectroscopy. For the NMR analysis, a liquid sample was prepared by mixing 500 μL of electrolyzed solution with 100 μL D2O and 100 μL of DMSO/H_2_O (1/1000) *v*/*v*) used as an internal standard.

#### 2.1.7. Preparation of H_2_ and C_2_H_4_ Calibration Curves

The steps involved in the construction of standard calibration curves of the gaseous products H_2_ and C_2_H_4_ are as follows

1. Electrolyte solution and head space were saturated with pure CO_2_ into the same H-cell that was used for bulk electrolysis;
2. Standard gases of C_2_H_4_ and H_2_ of known amounts were injected into the electrolyte solution and head space of the same volume;
3. The mixture of gases of the same volume was then injected to GC to obtain the area of the peak corresponding to H_2_ and C_2_H_4_;
4. Range of different amounts of C_2_H_4_ and H_2_ were tested and repeated the experiments to obtain the calibration plots of C_2_H_4_ and H_2;_
5. Calibration curves of C_2_H_4_ and H_2_ were obtained by plotting the concentration of gases against the peak intensity of the respective gases.

#### 2.1.8. Faradaic Efficiency

Faradic efficiency of gaseous and liquid products was calculated from the following equation:

FE = znF/Q,

where z is the number of electrons required per molecule to obtain the respective product, F is the Faraday constant, n is the no. of moles of each product (i.e., obtained from the calibration plot) and Q is the total charge (C) consumed during bulk electrolysis.

Overpotential was calculated from the following equation:

η = |E − E^0′^|,

where E is the applied potential, η is the overpotential, and E^0′^ is the equilibrium potential for the reduction of CO_2_ to Ethylene and Ethanol.

## 3. Results and Discussion

### 3.1. Physical Characterization

**Figure 1:**
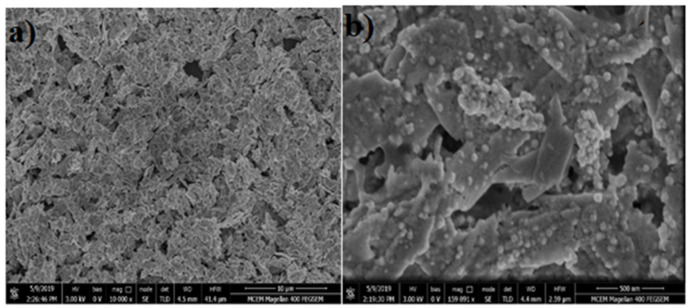
SEM image of CuO (**a**) before and (**b**) after electrochemical reduction at −0.95 V vs. RHE.

**Figure 2:**
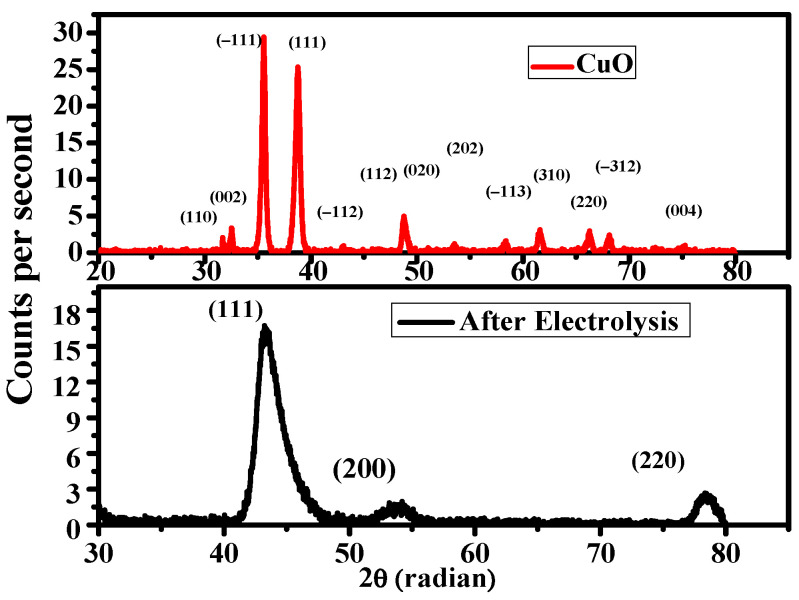
The X-ray diffraction pattern of CuO before and after electrochemical reduction at −0.95 V vs. RHE.

The oxidation state of CuO was reduced during CO_2_ reduction as evidenced from XRD. Cu 111 ions are believed to be an active species for reducing CO_2_ to C_2_ compounds [27,28]. It was observed that the surface of CuO, apparently in blue, reduced and converted to brick brown Cu NPs particles during ERC. SEM images were taken to show the surface morphology of CuO and Cu NPs. The SEM image (Figure 1a) shows a uniform coating of thick and dense copper oxide nanoparticles. After electrolysis, the SEM image (Figure 1b) displays a granule like Cu nanoparticles surface morphology with a size approximately in the range of 20–78 nm. The SEM image after CO_2_ reduction indicates the defect sites on the catalyst surface along with spherical Cu particles. In XRD (Figure 2), only Cu⁰ peaks can be seen in the XRD pattern after electrolysis. These results specify the complete reduction of CuO during electrocatalysis [27,28,29].

### 3.2. Cyclic Voltammetry

**Figure 3:**
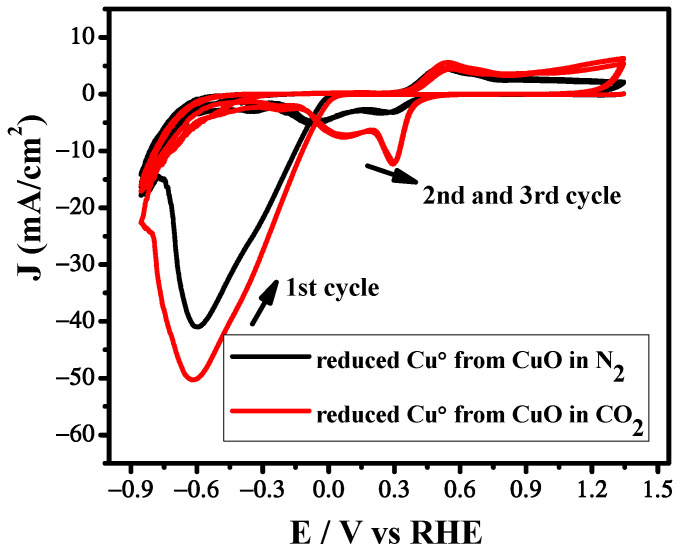
Cyclic voltammetry measurements under N_2_ and CO_2_ atmosphere using CuO catalyst. Scan rate: 100 mV s^−1^.

The electrochemical behavior of the Cu/CuO catalyst was firstly investigated by cyclic voltammetry in N_2_ and CO_2_ saturated 5 M KHCO_3_ electrolyte using a three electrode system, a glassy carbon electrode as a working electrode, Ag/AgCl as a reference electrode and Pt as a counter electrode at a scan rate of 100 mV/s. Cyclic voltammetric behavior of the Cu/CuO catalyst in N_2_ atmosphere is different than that of the Cu/CuO catalyst in CO_2_ atmosphere, as shown in Figure 3. The higher current in N_2_ atmosphere can be attributed to the hydrogen evolution reaction. However, the rapid increase of current in CO_2_ atmosphere as compared to N_2_ suggests that the CO_2_ reduction is catalytically more favorable than HER [30].

In the first scan cyclic voltammogram, a reduction peak was observed around 0.6 V after purging with N_2_ and CO_2_, followed by the successive two oxidation signals appearing at 0.52 V and 0.65 V during the reverse scan. These two oxidation peaks also appeared in the successive scan. The first oxidative peak corresponds to the oxidation of Cu to Cu(I) and the second oxidation peak may be due to the conversion of Cu(I) to CuO as reported by the previous investigators [31,32]. During the second scan of cyclic voltammograms two reductive peaks were observed at 0.30 V and 0.069 V, respectively, which can be due to the conversion of CuO to Cu(I) and the transition of Cu(I) to Cu. Moreover, the disappearance of peak I in the subsequent scan can be attributed to the combination of reduction of the CuO to Cu^+^ and Cu at more negative potentials. Therefore, we suggest that the reduction peak observed in the 1st scan is the reduction of CuO to Cu. The catalyst attained a more stable structure as most of the CuO was already converted to Cu^+^ and Cu. Therefore, after the 1st scan, all the subsequent scans had a similar pattern of reduction peaks. The trend of the catalyst in N_2_ and CO_2_ saturated KHCO_3_ is almost similar; however, the shifting of peaks and their intensification can be attributed to the CO_2_ reduction on the electrode surface.

**Figure 4:**
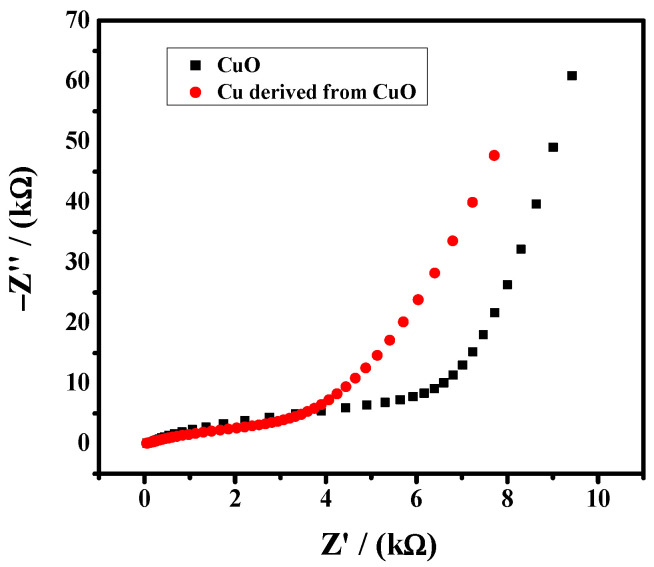
EIS spectra of catalyst in 5 mM K_3_Fe(CN)_6_ solution. Frequency range is from 1 Hz to 14 kHz.

The electrochemical activity of the CuO and reduced Cu was evaluated by EIS. As shown in Figure 4, reduced copper exhibits a better charge transfer rate as the reduced copper nanoparticles have a high electrochemical surface area which offers more active sites.

### 3.3. Bulk Electrolysis

**Figure 5:**
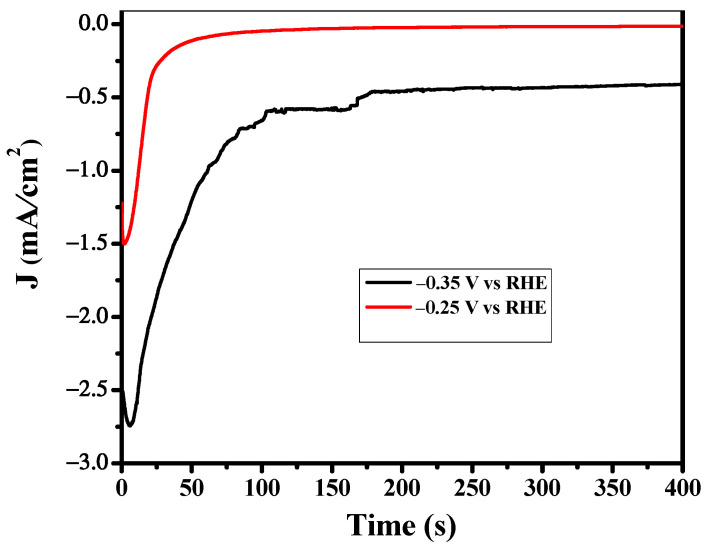
Reduction current density of CO_2_ as a function of time. Electrolyte: 0.5 M KHCO_3_.

Bulk electrolysis was carried out in a sealed H-cell to investigate the CO_2_RR performance of the Cu derived CuO catalyst. Ethene, Ethanol and H_2_ were the dominant reduction products accompanied by propanol and formic acid under applied potential. The formation of ethanol and ethene occurs at 0.10 V vs. RHE with a lower F.E but still on the lowest overpotential. This confirms that the surface morphology of the catalyst plays a significant role in C_2_ product formation. At the highest negative potential, the Faradic efficiency of C_2+_ is significantly high as the values of F.E of ethylene, ethanol and propanol are 20%, 33% and 4% respectively at −0.95 V vs. RHE. So, overall, the F.E of C_2+_ products is 57%, which is remarkably high as compared to the 14% Faradic efficiency of C1 product i.e., formic acid and 29% Faradic efficiency of H_2_. Statistically, CuO derived Cu plays a significant role in C_2_ product formation. This may be due to the origin of the structural transformation of the oxides of Cu to Cu during CO_2_ reduction as confirmed by XRD and SEM results. Hence, the transformation induces a defective site in the catalytic surface which is believed to play a crucial role in C_2_ products formation even at the lowest overpotential. CO_2_ reduction was performed using the CuO derived Cu catalyst at a fixed potential between −0.1 V to −1.5 V vs. RHE. Figure 5 shows the plot of current vs. time during CO_2_ reduction at the potential of −0.35 V and −0.25 V. A reduction peak observed during the initial phase of the curve represents the reduction of CuO to Cu. A similar trend was observed in all amperograms at all applied potentials. This feature is consistent with the XRD result.

### 3.4. Effect of Electrolyte

Alkali-metal ions favor CO_2_ adsorption and are preferentially used in CO_2_RR. These cations stabilize the surface intermediates by creating a field effect or by interacting with the adsorbed species. Moreover, the overpotential of the catalyst decreases with the increase of cationic size. In addition, the current density also increases with the increase of cationic size, hence favors more C_2_ products as compared to C_1_. In our study, Cu 111 in KHCO_3_ showed more Faradic efficiency for C_2_ products as compared to Cu 111 in NaHCO_3_. This study confirms that multivalent cations used as supporting electrolytes influence the rate of CO_2_RR. The possible explanation may be due to the preferential hydrolysis or the greater capacity for specific adsorption [32,33,34,35,36].

Product distribution of CO_2_ reduction mainly depends upon the type of crystal facets. CO_2_ adsorption depends upon the surface orientation of crystals as its variation affects the Lewis acidity and polarizability of CO_2_. Facets which decrease activation energy and increase the adsorption of CO_2_ are more feasible for CO_2_. In our study, XRD results reveal that the formation of Cu (111) from CuO favors the C_2_ products over C_1,_ suggesting the dimerization of CO that leads to the production of ethylene and alcohols [36,37,38,39].

**Figure 6:**
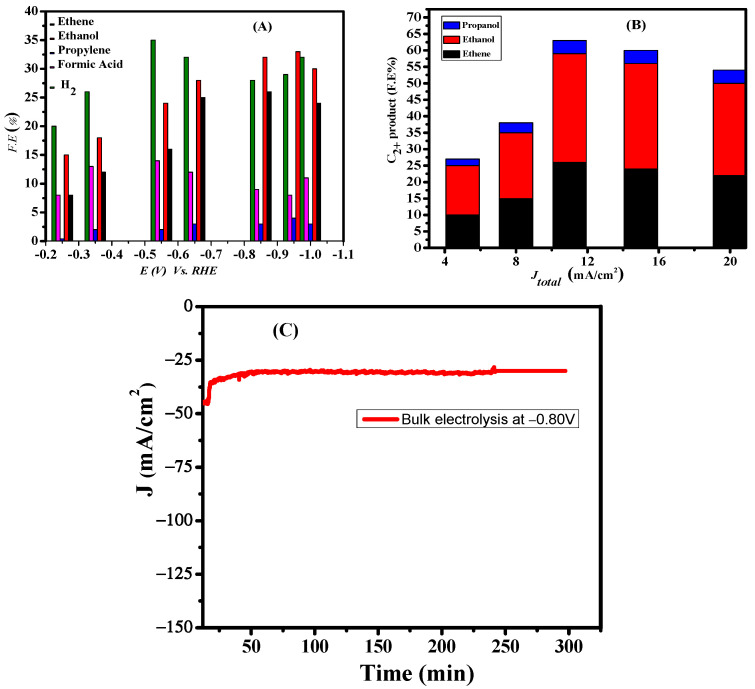
(**A**) CO_2_ reduction Faradic efficiency as a function of potential; (**B**) Plot of log J vs. potential Faradaic efficiencies of C_2+_ (ethene, ethanol and propanol) on Cu Nano catalyst in the current density range of 10–70 mA/cm^2^. Electrolyte: 0.5 M KHCO_3_; (**C**) Chronoamperometry results at −0.8 V.

A catalytic approach towards the formation of products with different Faradic efficiencies at various applied potentials (from −0.10 to −1.5 V) was studied thoroughly as shown in Figure 6A. The Cu 111 derived from CuO delivered a remarkable result with excellent F.E of hydrocarbons at the lowest overpotential for the catalytic selectivity towards the formation of C_2+_ products over C_1_. These results show that C_2+_ product is the dominant product, and that the selectivity is strongly dependent on the applied potential, current density and the catalytic activity of the electrode. At an applied potential of −0.20 V, C_2_ products are formed with F.E of 8% ethylene and 15% ethanol. Thus, the intrinsic property of catalyst favors multi-carbon products formation even at the lowest overpotential. During CO_2_RR, the FE of H_2_ was found in the range of 20% to 36%. Another impressive result at the lowest overpotential is the formation of n-propanol with 4% Faradic efficiency.

The total current density versus maximum C_2+_ alcohol Faradaic efficiency is presented in Figure 6B. The current densities ranged from 10 to 70 mA/cm^2^ depending on the applied potential. The optimal potential for C_2+_ products (ethylene ethanol and propanol) was −0.95 V versus RHE with a peak FE of 57%. Beyond this potential, a decrease in C_2_ FE is observed, whereas the FE of H_2_ increases. This may be due to increased current density which produces larger bubbles inducing cracks in the catalyst surface and thus exposes the carbon plate surface. This exposed carbon surface facilitates hydrogen production and thus a decrease in the Faradic efficiency of ethylene is observed.

To probe the durability of the CuO catalyst, electrolysis was carried out by chronopotentiometry at −0.80 V. The current density was stable at about −30 mA cm^−2^ over 5 h as shown in Figure 6C. A comparison of the reported Faradaic efficiency of C_2_ products on various copper surfaces with our catalyst can be seen in Table 1. The listed data reveal that the surface morphology of the catalyst affects the overpotential and selectivity of the CO2RR product. The Faradaic efficiency of ODCu for the C_2+_ product formation is higher than that in the reported literature [38,39,40,41,42,43,44,45,46] cited in Table 1. Moreover, one of the remarkable results of our designed catalyst is its onset potential of −0.1 V for the C_2_ products formation, which is the lowest overpotential amongst the reported works. Another figure of merit of our catalyst is its higher current density at lower overpotentials.

## 4. Origin of Selectivity

**Figure 7:**
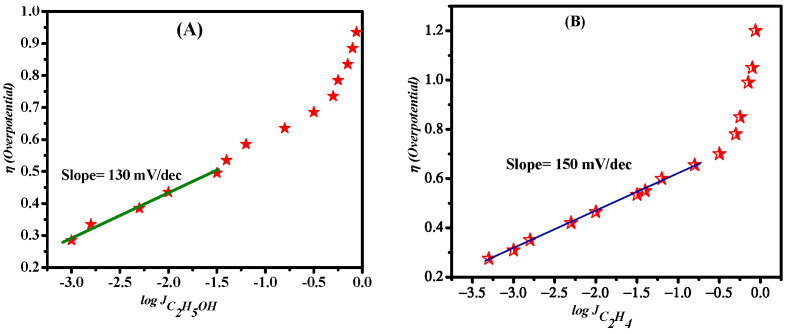
(**A**) Tafel plot for ethanol (**B**) Tafel plot for ethylene.

To obtain mechanistic insights about the kinetics of RDS of the reaction on the catalyst, a Tafel plot analysis was carried out. This analysis is applicable only when the reaction is kinetically controlled [4,5,6,7,8]. Interestingly, we attained the C_2_ products at a lower overpotential owing to the excellent intrinsic property of the catalyst. The corresponding Tafel slopes for C_2_H_4_ and C_2_H_5_OH are shown in Figure 7. The plot of C_2_H_4_ production exhibited a lower Tafel slope of 124 mV dec^−1^ than that of EtOH (150 mV dec^−1^). Both plots are close to the value of 118 mV dec^−1^, which indicates that C_2_H_4_ and EtOH involve a common intermediate with a rate determining single electron transfer to CO_2_. For ethanol and ethylene, Tafel plots are linear at a lower potentials range which is mainly a kinetically controlled region while the higher potential region is controlled by the mass transportation of ions and diffusion of gases. Due to this combined effect, a steeper slope is obtained at a higher potential region. During CO_2_ reduction, CuO reduces to Cu, hence stepped edges and grain boundaries are expected to be formed on the catalytic surface. In the literature, temperature-controlled desorption studies and density functional theory calculations show that CO adsorbs more strongly on Cu edges and grains as compared to the flat surfaces. Step edges and grain boundaries are suggested to have highly active sites that favor C–C coupling, as they offer lower energy barriers for the formation of key intermediates (CO, CHO etc.) [1,2,3,4,5,40,41,42,43,44,45,46,47,48].

**Figure 8:**
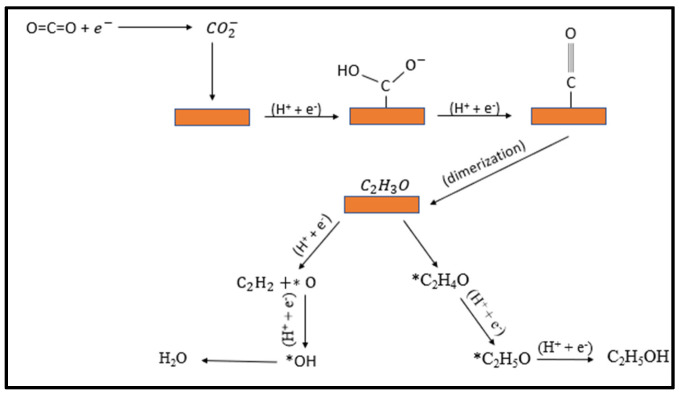
Proposed mechanism for the electroreduction of CO_2_ to ethylene and ethanol on copper surfaces.

CO_2_ electroreduction on a copper surface results in many C_1_ and multi-carbon products including CH_4_, CO, HCOOH, C_2_H_4_, C_2_H_5_OH etc. In the CO2RR process, generally one electron transfer to CO_2_ is considered as the rate determining step, i.e., the formation of *CO_2_^•−^. Cu can convert CO_2_ to methane, ethylene and ethanol with high Faradaic efficiency owing to a strong bonding propensity of Cu with *CO_2_^•−^ intermediate, which reduces CO further into higher order hydrocarbons. Complex processes involved in CO_2_ reduction to C_2+_ products with several possible pathways are still the focus of substantial theoretical study. Density functional theory (DFT) calculations using the CHE (computational hydrogen electrode) model propose that a potential-limiting step for the hydrocarbon’s formation is the protonation of CO* to CHO* on the surface of Cu [49]. A number of pathways have been proposed that suggest the formation of intermediate hydroxyl–methylidyne and the dimerization of two CO* molecules [50]. Current advancement in computational studies further helps in understanding the mechanism and suggests that *CHO or *COH intermediate formation from the hydrogenation of *CO also play a pivotal role in determining the favorable products such as C_2_H_4_, CH_4_, and CH_3_OH on a Cu (111) surface [51]. This kinetically challenging step is due to the high energy barrier required to drive the reaction. Ethanol and ethylene as a multi-carbon product require coupling of C–C between the adsorbed intermediates on the surface, so the dimerization steps favor multi-carbon products. Recent theoretical calculations on the Cu (111) surfaces suggest that the C–C coupling strongly depends on the degree of CO_ads_ and hydrogenation and that energy barrier of coupling decreases with the increase of the hydrogenation of surface bounded CO, which tends to favor C_2_ product formation. According to the Tafel plot, the reduction of CO_2_ is the rate determining step, so that adsorbed intermediate CO_2_^−^ must be stable enough to persist until the availability of other intermediates for coupling. Copper nanocrystals may stabilize C_1_ and C_2_ intermediates, allowing them to trimerize to a C_3_ compound (n propanol). The reaction mechanism involves various steps and the formation of several products [3] as shown in Figure 8.

## 5. Conclusions

In summary, CuO derived Cu was prepared, which demonstrated an efficient electrocatalytic performance for carbon dioxide reduction in aqueous media. The oxide derived Cu reduced to metallic copper during CO_2_RR induced defective sites and that transformation facilitated C_2_ products. C_2+_ products were obtained at a higher FE mainly consisting of ethylene, ethanol and propanol at −0.95 V. The catalyst was found to exhibit long term stability with a current density of almost −30 mAcm^−2^. The remarkable finding of this catalyst is its onset potential of −0.1 V for C_2_ product which is quite a low overpotential for C_2_ products formation. The intrinsic property of the catalyst and catalyst surface reduction may induce sites or edges that facilitate the dimerization and coupling of intermediates to ethanol and ethylene. Further research is still required to ensure the mechanistic pathway. In this regard, DFT calculations can be helpful to determine the formation of possible intermediates on the surface of the catalyst and the possible interaction of the intermediates on the catalyst surface that stabilizes the C_2_H_3_O. It can also be possible through DFT calculations to calculate the energy barrier required for the formation of CO_2_^−^ and for the key step involved in the C–C coupling leading to the formation of C_2_ products at the lowest overpotential. There is an ongoing debate about the nature of C–C coupling and whether it is a chemical step or an electrochemical step that involves the transfer of H^+^/e for the formation of C_2_ products at lower overpotentials.

## Figures and Tables

**Table 1:** Comparison of reported Faradaic efficiency of C_2_ products on various copper surfaces with the proposed catalyst.

Catalyst	Experimental Condition	Onset Potential	Products	Faradic Efficiency	Refs.
Copper oxide derived catalyst	0.2 M KHCO_3_ @ −1.6 V (vs. RHE)	N/A	C_2_H_4_	29.7%	[38]
Cu-porphyrin complex	−0.976 V (vs. RHE)	−0.976 V (vs. RHE)	C_2_H_4_	17%	[46]
Cu skeletons	0.5 M NaHCO_3_ −1.1 V (vs. RHE)	−0.25 V vs. RHE	C_2+_ products:	32.2%	[39]
Cu NWs	0.1 M KHCO_3_ −1.1 V (vs. RHE)	N/A	C_2_H_4_	17.4%	[44]
Cu meshes	0.5 M KHCO_3_ @ −1.1 V (vs. RHE)	−0.7 V (vs RHE)	C_2_H_4_	34.3%	[40]
Cu/C_3_N_4_	~7.5 mA/cm^2^ @ −1.6 V (vs. Ag/AgCl)	−0.75 V vs. RHE	C_2_H_4_	~18%	[47]
Nanoporous Cu film	14.3 mA/cm^2^ −1.7 V (vs. NHE)	−0.96 V vs. NHE	C_2_H_6_	46%	[45]
Cu(II) Phthalocyanine/C	2.8 mA/cm^2^ @−1.6 V (vs. Ag/AgCl)	N/A	C_2_H_4_: 25%	25%	[43]
Cu/MoS_2_	0.1 M KHCO_3_	N/A	C_2_H_5_OH	42.4%	[41]
Cu nanocube	0.25 M KHCO_3_ 68 mA/cm^2^ @ 0.963 V	−0.7 V (vs. RHE)	C_2_H_4_	32%	[42]
ODCu	−0.95 V vs. RHE	−0.10 vs. RHE	C_2+_ products Ethylene Ethanol Propanol	57% 20% 33% 4%	Present work

## Data Availability

Not applicable.
